# Superoxide Mediates Direct Current Electric Field-Induced Directional Migration of Glioma Cells through the Activation of AKT and ERK

**DOI:** 10.1371/journal.pone.0061195

**Published:** 2013-04-16

**Authors:** Fei Li, Tunan Chen, Shengli Hu, Jiangkai Lin, Rong Hu, Hua Feng

**Affiliations:** Department of Neurosurgery, Southwest Hospital, Third Military Medical University, Chongqing, China; University of Illinois College of Medicine, United States of America

## Abstract

Direct current electric fields (DCEFs) can induce directional migration for many cell types through activation of intracellular signaling pathways. However, the mechanisms that bridge extracellular electrical stimulation with intracellular signaling remain largely unknown. In the current study, we found that a DCEF can induce the directional migration of U87, C6 and U251 glioma cells to the cathode and stimulate the production of hydrogen peroxide and superoxide. Subsequent studies demonstrated that the electrotaxis of glioma cells were abolished by the superoxide inhibitor N-acetyl-l-cysteine (NAC) or overexpression of mitochondrial superoxide dismutase (MnSOD), but was not affected by inhibition of hydrogen peroxide through the overexpression of catalase. Furthermore, we found that the presence of NAC, as well as the overexpression of MnSOD, could almost completely abolish the activation of Akt, extracellular-signal-regulated kinase (Erk)1/2, c-Jun N-terminal kinase (JNK), and p38, although only JNK and p38 were affected by overexpression of catalase. The presenting of specific inhibitors can decrease the activation of Erk1/2 or Akt as well as the directional migration of glioma cells. Collectively, our data demonstrate that superoxide may play a critical role in DCEF-induced directional migration of glioma cells through the regulation of Akt and Erk1/2 activation. This study provides novel evidence that the superoxide is at least one of the “bridges” coupling the extracellular electric stimulation to the intracellular signals during DCEF-mediated cell directional migration.

## Introduction

Electrotaxis is defined as the directional movement of cells towards the cathode or anode under an electric field. The migration of living cells in a direct current electric field (DCEF) was discovered many years ago [1], and has been observed in several cell types [2], [3], [4], [5], [6], [7], [8], [9]. Endogenous electric fields, which have strengths of 10–30 mV and generate an electric field of 200–600 mV/mm, are thought to play a role in development, regeneration, and wound healing. In addition, it has been well established that DCEF plays a crucial role in neurogenesis, axon guidance, and nerve growth in the nervous system. Early in development, the creation of the nervous system requires the presence of an electric field [10], and an electric field as low as 100 mV/mm can cause growth cones to turn, usually toward the cathode [10]. Electric fields are induced in damaged axons, and these injury-induced electric fields are believed to contribute to axonal regeneration. The application of DCEF in rat brain injury models has been shown to induce functional improvements [11], [12]. Although the concept of electrotaxis, the systems for *in vitro* observation, and the factors involved in these processes, including ion channels, cell membrane, intracellular signals, and cytoskeletons, have been well documented as being involved in electrotaxis [4], [13], [14], [15], the mechanisms underlying their roles have not been elucidated.

Gliomas are the most common primary brain tumors. Although great progress has been made in glioma treatment in the past few decades, the prognosis of patients with malignant gliomas is still poor [16]. The median overall survival of patients with high-grade glioma, even after surgery, radiotherapy, and chemotherapy, is approximately 22 months for anaplastic astrocytoma and 16 months for glioblastoma [17]. The histological feature of malignant gliomas is the invasion of tumor cells in surrounding normal brain tissue. Glioma cells preferentially invade along the fibers in white matter tracts, and the invasion of intrafascicular, subpial, periventricular, and intra-corpus callosum regions by glioma cells is frequently encountered in patients [18]. These white matter fibers, which mainly function in transmitting electrical signals, generate electric fields around the axis. Moreover, epileptic seizures are a frequent clinical manifestation of cerebral glioma and complicate the clinical course in more than 80% of these patients [19]. Abnormal discharges, which are much higher than physiological electrical signals, also spread from the nest of the tumor to distant regions through white matter fibers. Although electrotactic responses of cancer cells have been reported in recent years [5], [20], [21], it remains unknown if the migration of glioma cells is affected by the electric field around them, or if the gradients of electric fields provide some guidance cues for glioma cell invasion of normal brain tissue.

There is growing evidence showing a correlation between reactive oxygen species (ROS) and directional cell migration. ROS have been identified as key regulators of neutrophil chemotactic migration [22], hepatic pro-fibrogenic cells [23], and breast cancer cells [24]. In this study, we examined whether DCEF could exert effects on glioma migration, and then determined how ROS and intracellular signals are involved in mediating DCEF-induced glioma migration. We found that DCEF can direct and facilitate the migration of U87, U251, and C6 glioma cells towards the cathode and induce the generation of ROS. Furthermore, we showed that DCEF-induced ROS generation and directional migration are blocked by ROS scavengers or overexpression of mitochondrial superoxide dismutase (MnSOD), but not by overexpression of hydrogen peroxide catalases in the mitochondria (mCat). Finally, our studies showed that superoxide-activated phosphatidylinositol-3-kinase (PI3K) and mitogen-activated protein kinases (MAPK) signal transduction pathways may be involved in DCEF-induced directional cell migration.

## Materials and Methods

### Plasmids, Antibodies, and Other Reagents

The plasmid expressing MnSOD, empty vector, viral vector encoding mitochondrial Catalase (Ad-mCAT), and the control LacZ virus (Ad-LacZ) were gifts from Dr. Chuanshu Huang from the New York University School of Medicine. The antibodies against p-Akt, Akt, p-extracellular-signal-regulated kinase p-Erk1/2, Erk, p-c-Jun N-terminal kinase (JNK), JNK, p-p38, p38, SOD, and catalase (CAT) were purchased from Cell Signaling Technology, Inc. (Boston, MA, USA). The anti-ß-actin antibody, N-acetyl-l-cysteine (NAC), PD98059 and LY294002 were obtained from Sigma-Aldrich (St. Louis, MO. USA). Dichlorfluorescein-diacetate (DCFH-DA), hydroethidine (HE) and Alexa Fluor 488 conjugated goat anti-rabbit IgG were purchased from Invitrogen (Carlsbad, CA, USA). The Cytoskeleton II Phosphorylation Antibody Array was purchased from Full Moon BioSystems (Sunnyvale, CA, USA).

### Cell Culture and Transfection

The human U87 MG and U251 glioma cell lines were obtained from the American Type Culture Collection (Manassas, VA, USA), the rat C6 glioma and mouse astrocyte cell lines were obtained from the Cell Bank of the Chinese Academy of Sciences (Shanghai, China). The cells were maintained at 37°C in a 5% CO2 incubator in Dulbecco’s modified Eagle’s medium (DMEM) with 10% fetal bovine serum (FBS), 2 mM L-glutamine, and 25 µg/ml gentamicin. Cells were trypsinized at 80% confluence in tissue culture dishes, collected, and then seeded onto Falcon-faced coverslips placed in 24-well plates. The cells were further incubated for 24 h (37°C, 5% CO_2_) before DCEFs were applied. The human U87 MG cell transfections were performed with the Polyjet™ *in vitro* DNA Transfection Regent (SignaGen, Gaithersburg, MD, USA) according to the manufacturer’s instructions.

### ROS Detection by Luminescence and fluorescence Microscopy Observations

The production of ROS was monitored by the SpectraMAX M2 spectrophotometer (Molecular Devices Corp, Sunnyvale, CA, USA) using DCFH-DA and HE. The fluorescence intensity is proportional to the amount of ROS produced by the cells. DCFH-DA is a well-established compound used to detect and quantify intracellular-produced hydrogen peroxide (H_2_O_2_) [25]. The other probe, HE, is oxidized rapidly to the fluorescent molecule, ethidium bromide, by superoxide (O_2_^•−^) and is considered a good indicator of intracellular O_2_^•−^ [26]. In the present study, DCHF-DA (10 µM) and HE (10 µM) were added to the cell cultures 30 min before treating the cells with the indicated DCEF. DCEF-treated cells were then washed twice with phosphate-buffered saline (PBS) to remove the extracellular compounds, and the fluorescence intensities were measured by a SpectraMAX M2 spectrophotometer with excitation of 488 nm and emission of 530 nm for DCHF-DA and excitation of 510 nm and emission of 600 nm for HE. The fluorescence of cells was also observed and images were taken under a fluorescence microscope.

### DCEF Application and the Imaging of Cell Migration

Chambers were selected for observation as previously described [14], [27]. For DCEF application, agar-salt bridges were used to connect silver/silver chloride electrodes in beakers of Steinberg’s solution (60 mM NaCl, 0.7 mM KCl, 0.8 mM MgSO_4_·7H_2_O, 0.3 mM CaNO_3_·4H_2_O, 1.4 mM Tris base, pH 7.4) to pools of excess culture medium on either side of the chamber. Field strengths were measured directly at the beginning and end of the observation period. For time-lapse observations, HEPES acid (25 mM) was added to the culture medium and the pH was adjusted to 7.4 Field strengths of 100, 200, 300, and 250 mV/mm were used for these experiments, with an exposure time of 90 to 180 min. Time-lapse imaging was performed with a Leica 6000B microscope (Leica Microsystems Inc., Buffalo Grove, IL, USA) that was used to digitally record cell migration, and images were acquired every three minutes. The cultures and the electric field (EF) stimulation device were maintained in the CO_2_ incubator at 37°C.

### Quantification of Cell Motion

To quantify the velocity and direction of cell motion in the time-lapse experiments, the cells centroids were calculated with ImageJ software (NIH) [7]. This calculation yielded (x, y) coordinates in micrometers. Absolute motion in the x- and y-planes was used to calculate displacement for each cell. Displacement was divided by the duration of the observation to yield velocity in micrometers per hour. The direction of motion was expressed as a function of cosine, and was calculated by defining movements in the x-plane toward the cathode (or mock-cathode) as positive. This was divided by displacement to yield the direction for each cell. Therefore, a cell moving directly toward the cathode would have a value of +1, whereas a cell moving directly toward the anode would have a value of −1.

### Western Blot Analysis

Cell extracts were prepared by incubating the cells in lysis buffer containing 10 mM Tris-HCl, pH 7.4, 1% SDS, and 1 mM Na_3_VO_4_ for 15 min on ice. Protein concentrations were determined using a NanoDrop 2000 (Thermo Fisher Scientific Inc, Waltham, MA, USA). Cytoskeleton II Phosphorylation Antibody Arrays were analyzed by Sangon Biotech Co., Ltd (Shanghai, China). Fifty micrograms of protein were subjected to a Western blot analysis as described in previous studies [28].

### Immunofluorescence

U87 and U251 cells were plated onto coverslips and maintained in culture. At 50% confluence, the cells were fixed in 3.7% paraformaldehyde. After washing with cold PBS containing 0.1% Triton X-100, the cells were incubated with 2% goat serum in PBS to block nonspecific binding. The fixed cells were treated with a rabbit polyclonal anti-human p-Akt and p-Erk1/2 antibody at 1∶200 dilution overnight at 4°C. The cells were then incubated with a Alexa Fluor 488 conjugated goat anti-rabbit immunoglobulin G at 1∶200 dilution for 1 hour in the dark. Images were acquired using a Leica 6000B microscope.

### Statistical Methods

The Student’s t-test was used to determine the significance of difference in cell migration and ROS generation among various treatments using SPSS software (SPSS, Inc., Chicago, IL, USA). In addition, two-way ANOVA was used to compare both groups and treatments within groups. Differences were considered to be significant at *P*<0.05.

## Results

### DCEF Induction of Directional Migration of U87, C6 and U251 Glioma Cells

**Figure 1:**
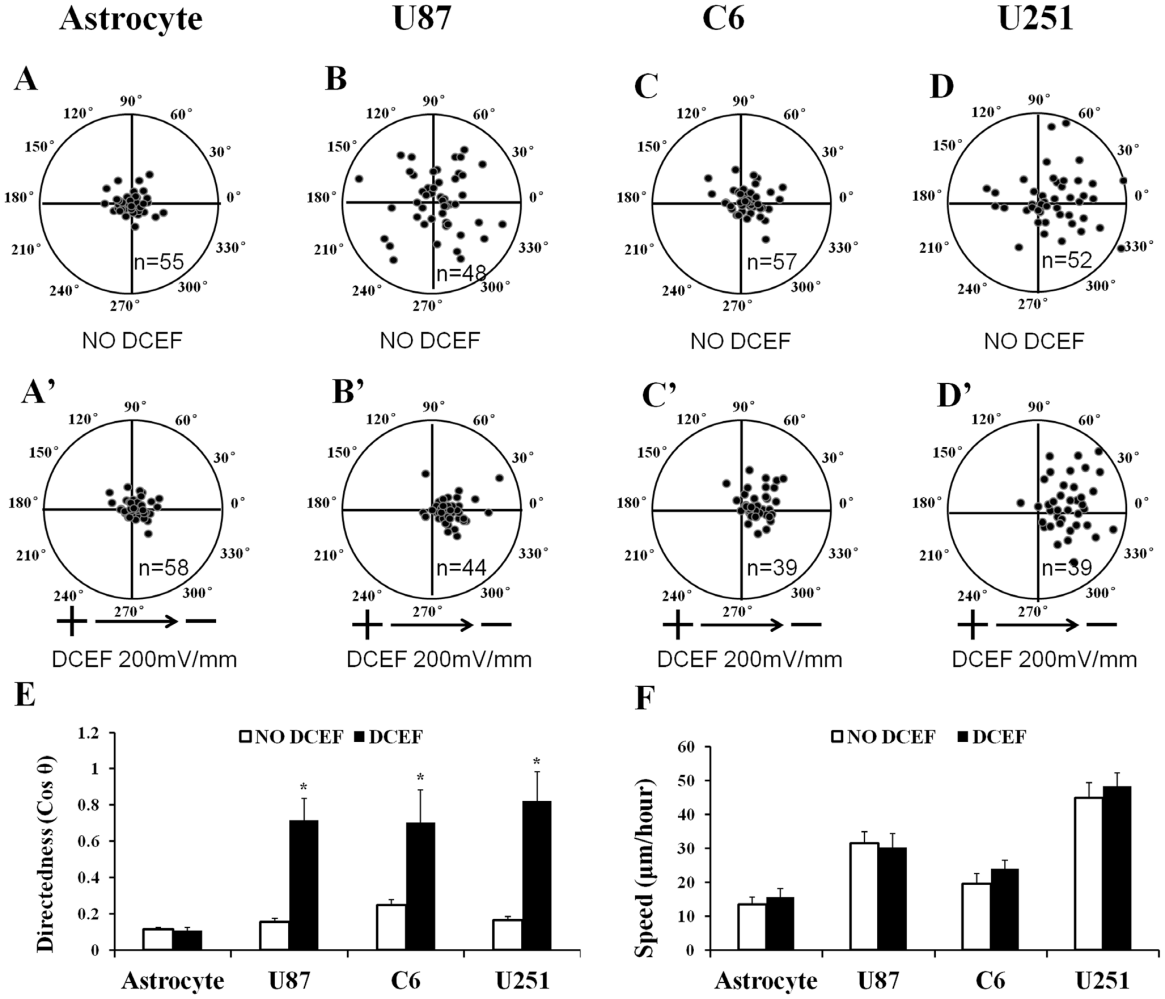
DCEF-mediated induction of directional migration of glioma cells. In the absence of DCEF, Astrocytes (A), U87 glioma cells (B), C6 glioma cells (C) and U251 glioma cells (D) migrated with equal probability in all directions. The DCEF in a 200 mV/mm could not direct the migration of the astrocyte (A′). Glioma cells migrated toward the cathode in a 200 mV/mm DCEF after two hours of exposure (B′–D′). An analysis of direction (cosθ) and speed (µm/h) of glioma cells migration (E and F). *P<0.05.

DCEF-induced directional movement of cells has been observed in many cell types [3], [4], [5], [6], [7], [9], [10], [12], [14], [15], [21], [27], [29]. In most cases, cells such as bovine aortic vascular endothelial cells, human keratinocytes, and mouse embryonic fibroblasts migrate towards the cathode [14], [30], but in some cell types, including human granulocytes, rabbit corneal endothelial cells, and human vascular endothelial cells, migration occurs towards the anode [27], [31]. Therefore, we assessed the migration of individual U87, C6, and U251 glioma and astrocyte cells within a DCEF. In the absence of an applied direct current electric field, astrocyte and glioma cells migrated with equal probability in all directions, with average cosine θ of 0.12±0.01 (astrocyte, Fig. 1A and E), 0.16±0.02 (U87, Fig. 1B), 0.25±0.03 (C6, Fig. 1C and E) and 0.17±0.02 (U251, Fig. 1D and E) respectively. In contrast, a 200 mV/mm DCEF significantly directed the migration of glioma cells toward the cathode (Fig. 1B and E), with average cosine θ of 0.72±0.12 (U87, Fig. 1B′ and E), 0.70±0.18 (C6, Fig. 1C′ and E) and 0.82±0.16 (U251, Fig. 1D′ and E) respectively. However, the DCEF could not direct the migration of the astrocyte with an average cosine θ of 0.11±0.02 (Fig. 1A′ and E). The average movement speed, which reflected the migratory activity of cells, did not differ when cells with or without DCEF were compared (Fig. 1F).

### DCEF Induces Phosphorylation of Select Proteins in U87 Glioma Cells

The phosphorylation levels of 60 selected proteins related to cell migration were detected by a protein array. The results showed that the phosphorylation levels of 54 (90%) of the proteins were increased 30 minutes after the introduction of 200 mV/mm DCEF (Table 1). Moreover, the phosphorylation of 19 proteins increased by 1.5-fold compared to cells not exposed to DCEF, including CaMK1, Cortactin, Erk1/2, Ezrin, mitogen-activated protein kinase kinase 1 (MEK1), MEK2, Merlin, mitogen-activated protein kinase kinase 6 (MKK6), Paxillin, PI3K p85, Rac1/cdc42, and vasodilator-stimulated phosphoprotein (VASP).

**Table 1:** Phosphorylation levels of 60 selected proteins related to cell migration were detected by a Full Moon Cytoskeleton II Phosphorylation Antibody Array.

Proteins	Folds	Proteins	Folds
Actin Pan(a/b/g) (Ab-55/53)	1.02	MEK1(Ab-217)	1.87
Calmodulin (Ab-79/81)	1.02	MEK1(Ab-221)	1.52
CaMK1-a (Ab-177)	1.96	MEK1(Ab-291)	1.72
CaMK2-β/γ/δ(Ab-287)	1.17	MEK2 (Ab-394)	1.75
CaMK4 (Ab-196/200)	1.15	Merlin (Ab-10)	1.88
CaMKII (Ab-286)	1.06	Merlin (Ab-518)	2.05
Cofilin (Ab-3)	1.40	MKK3 (Ab-189)	1.14
Cortactin (Ab-421)	1.85	MKK3/MAP2K3 (Ab-222)	1.49
Cortactin (Ab-466)	1.32	MKK6 (Ab-207)	1.58
c-Raf (Ab-296)	0.98	MKK7/MAP2K7 (Ab-271)	1.20
c-RAF (Ab-43)	1.16	Myosin regulatory light chain 2 (Ab-18)	1.02
CrkII (Ab-221)	1.12	p130Cas (Ab-165)	1.11
Erk1-p44/42 MAP Kinase (Ab-202)	2.12	p130Cas (Ab-410)	1.16
Erk1-p44/42 MAP Kinase (Ab-204)	1.79	Paxillin (Ab-118)	1.52
Erk3 (Ab-189)	1.29	Paxillin (Ab-31)	1.14
Ezrin (Ab-353)	1.83	PI3-kinase p85-subunit α/γ(Ab-467/199)	1.69
Ezrin (Ab-478)	1.16	PKA CAT (Ab-197)	0.85
Ezrin (Ab-566)	1.47	PKCα(Ab-657)	0.87
FAK (Ab-397)	1.11	PKCα/β II (Ab-638)	1.16
FAK (Ab-407)	1.08	PLCβ3 (Ab-537)	1.11
FAK (Ab-576)	1.06	PLCβ(Ab-1105)	1.12
FAK (Ab-861)	0.98	Rac1/cdc42 (Ab-71)	2.11
FAK (Ab-910)	1.24	Rho/Rac GNE factor 2 (Ab-885)	1.07
FAK (Ab-925)	1.20	Src (Ab-418)	1.08
Filamin A (Ab-2152)	1.24	Src (Ab-529)	0.99
GRB2 (Ab-159)	1.09	Src (Ab-75)	1.27
GTPase activating protein (Ab-387)	1.28	VASP (Ab-157)	1.52
LIMK1 (Ab-508)	1.03	VASP (Ab-238)	1.79
MEK1 (Ab-286)	1.98	WASP (Ab-290)	1.00
MEK1 (Ab-298)	1.63	WAVE1 (Ab-125)	1.10

The results showed the folds of phosphorylation levels 30 min after the introduction of 200mV/mm DCEF than that without DCEF.

### DCEF Induces the Generation of ROS in U87, C6 and U251 Glioma Cells

**Figure 2:**
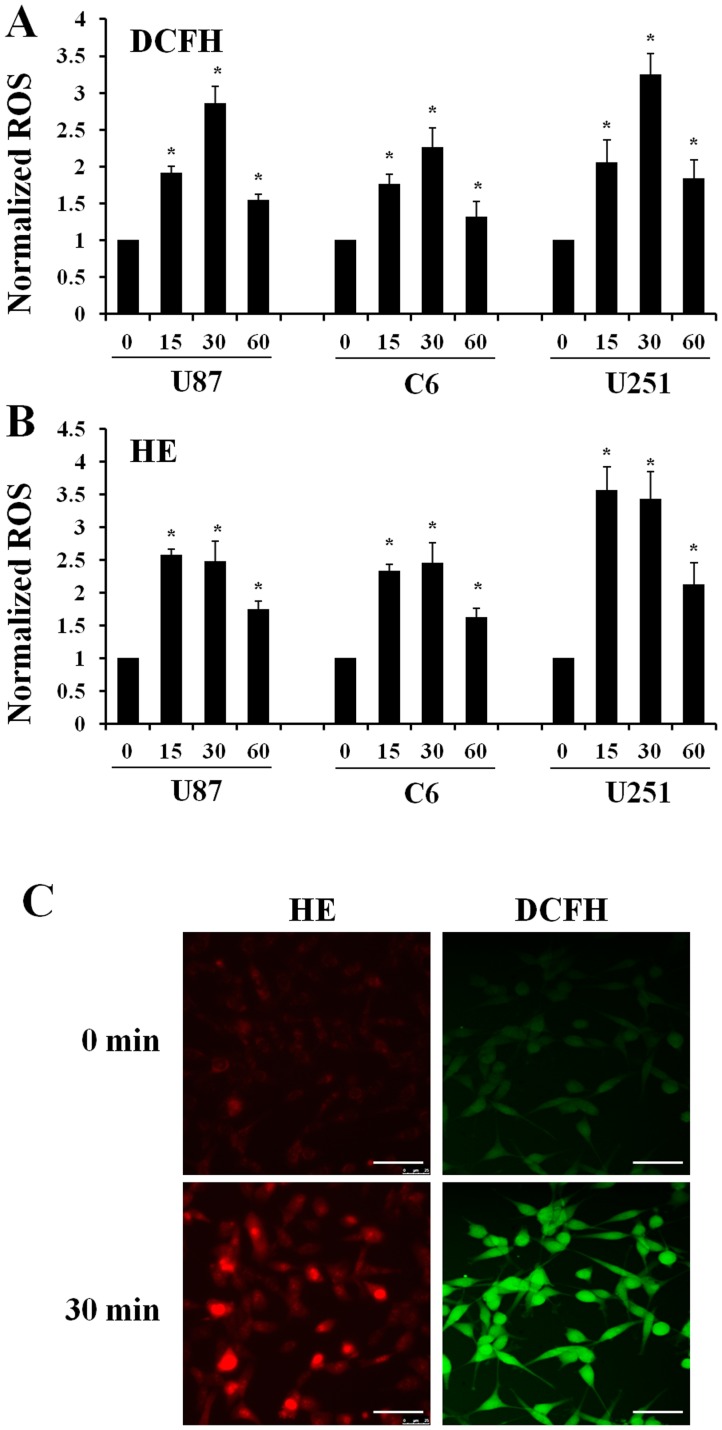
DCEF induces the generation of ROS in U87, C6 and U251 glioma cells. Generation of intracellular ROS was detected by the semi-quantitative technique based on the use of DCFH-DA (A) and HE (B) 15, 30, and 60 minutes after the treatment of cells with 200 mV/mm. Cells without DCEF stimulation were used as the negative control. Generation of intracellular hydrogen peroxide was detected by DCFH-DA (green fluorescence) and superoxide was detected by HE (red fluorescence) 30 minutes after EF treatment with a strength of 200 mV/mm (C). *Compared with the negative control, *P*<0.05; Bar = 50 µm.

Since MAPK and PI3K activation was observed after stimulating U87 cells with DCEF, and since ROS contribute to MnSOD-promoted migration/invasion in glioma cells through activation of Akt and Erk [32], we assessed ROS levels in this study. ROS has been showed to be involved in several cellular migratory processes, including wound repair, metastasis, and angiogenesis. However, the role of ROS in electrotaxis remains poorly understood. To investigate whether ROS are generated following electrical field treatment, U87, C6 and U251 cells were stained with the redox-sensitive dye DCFH-DA, which is a hydrogen peroxide (H_2_O_2_) detector, or hydroethidine (HE), which is a superoxide (O_2_^•−^) detector. The DCFH-DA fluorescence of DCFH significantly increased by up to 2.85±0.23-fold in U87 cells, 2.27±0.25-fold in C6 cells, and 3.25±0.28-fold in U251 cells exposed to a 200 mV/mm electric field, which peaked after 30 minutes of exposure, and remained higher than cells without DCEF after 60 minutes of exposure. These results indicate an increase in hydrogen peroxide after DCEF stimulation. In addition, HE fluorescence also increased by up to 2.57±0.12-fold and peaked at 15 minutes in U87 cells, 2.46±0.32-fold and peaked at 30 minutes in U87 cells, and 3.57±0.32-fold and peaked 15 minutes in U251 cells after DCEF stimulation, and remained higher than levels in cells not exposed to DCEF after 60 minutes of stimulation. These results indicated that superoxide levels increased after DCEF stimulation (Fig. 2A, B, and C).

### The Superoxide and Hydrogen Peroxide Scavenger NAC can Block DCEF-induced Directional Migration of U87, C6 and U251 Glioma Cells

**Figure 3:**
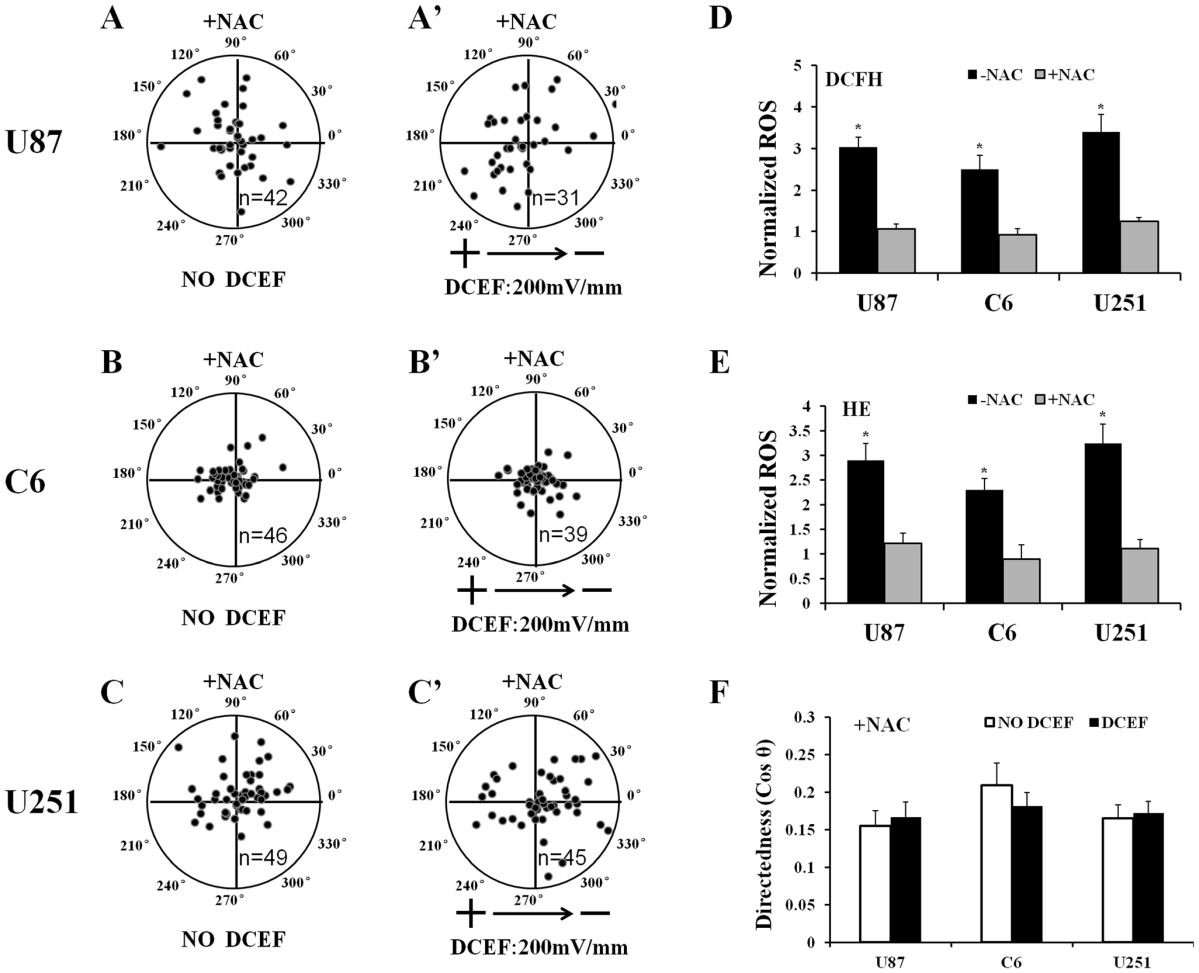
NAC blocks DCEF-induced directional migration of glioma cells. U87 (A and A′), C6 (B and B′) and U251 (C and C′) glioma cells migrate in all directions after two hours of NAC (10 mM) pretreatment with or without stimulation by a 200 mV/mm DCEF. ROS generation in cells stimulated with a 200 mV/mm DCEF for 30 minites with or without pretreatment with NAC (10 mM) as detected by DCFH-DA (D). Superoxide was detected by HE (E). Analysis of direction (cosθ) of U87 glioma cell migration (F). Data are expressed as the mean ± standard deviation (SD) (n = 3). * Compared with the NAC-pretreated cells, *P*<0.05.

We next performed experiments to investigate whether the intracellular generation of ROS may be critical in our experimental conditions. NAC (10 mM), which is a general pharmacological scavenger of ROS, was added to the cell culture 30 minutes before 200 mV/mm DCEF was applied. We found that pre-treating cells with NAC significantly inhibited or abolished both hydrogen peroxide and superoxide generation, as detected by DCFH and HE, in U87, C6 and U251 glioma cells respectively (Fig. 3D, E). The directional cathode migration of U87 (Fig. 3A, A′), C6 (Fig. 3B, B′) and U251 (Fig. 3C, C′) were also abolished by application of NAC (Fig. 3F).

### Superoxide, but not Hydrogen Peroxide, Responds to Electric Field-induced Directional Migration of U87 Glioma

**Figure 4:**
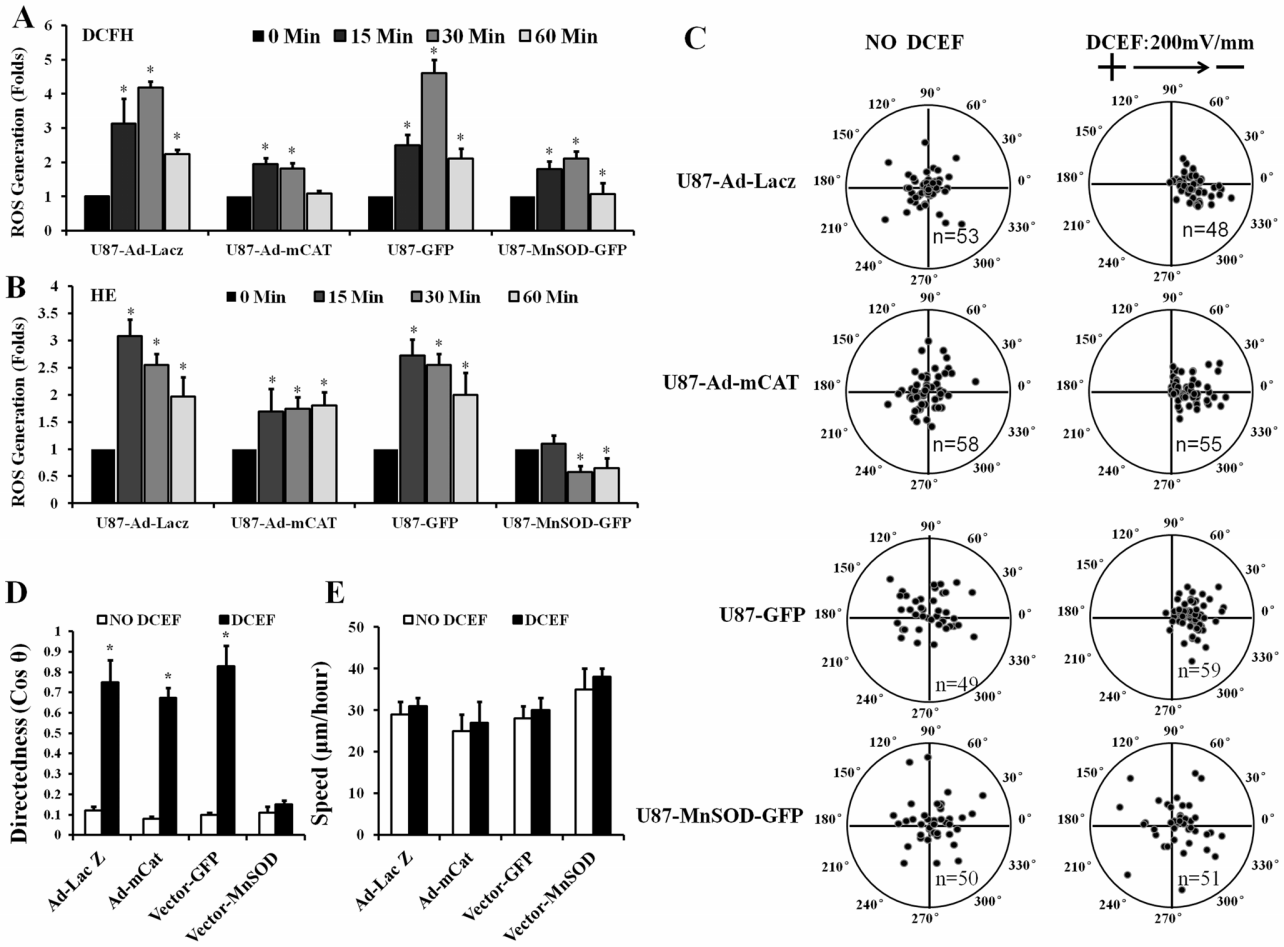
Overexpression of MnSOD, but not CAT, blocks DCEF-induced directional migration of U87 glioma cells. ROS generation in cells stimulated by an EF with a strength of 200 mV/mm 0, 15, 30, and 60 minutes with or without overexpression of MnSOD or mCAT, as detected by DCFH-DA (A) or HE (B). DCEF-induced (200 mV/mm) directional migration of U87 glioma cells is not affected by the overexpression of CAT, but is almost completely abolished by the overexpression of MnSOD (C). An analysis of direction (cos θ) and speed (µm/h) of U87 glioma cell migration (D and E). Data are expressed as the mean ± standard deviation (SD) (n = 3). *Compared to cells not receiving EF treatment, *P*<0.05.

NAC scavenged both hydrogen peroxide and superoxide, and abolished EF-mediated directional migration of glioma cells. To identify the specific effects of ROS in U87 cells, MnSOD, which specifically transforms superoxide into hydrogen peroxide and diatomic oxygen, and CAT, which specifically catalyzes the decomposition of hydrogen peroxide to water and oxygen, were overexpressed in cells. The results showed that expression of MnSOD and CAT decreased the electric field-induced generation of hydrogen peroxide, as indicated by the reduction in DCFH fluorescence (Fig. 4A). However, hydrogen peroxide levels increased by at least 2-fold after stimulation by EF (Fig. 4A). Similarly, the elevation of superoxide was partially inhibited by overexpression of CAT (Fig. 4B). In contrast, EF-induced generation of superoxide was totally abolished by overexpression of MnSOD (Fig. 4B). Accordingly, the directional migration of U87 cells was completely abolished by overexpression of MnSOD, but not CAT (Fig. 4C–4E). Importantly, the migration speed of U87 cells was not affected by overexpression of MnSOD or CAT (Fig. 4E). These results indicated that superoxide, but not hydrogen peroxide, plays a role in EF-induced directional migration.

### Activation of Akt and Erk 1/2 by Superoxide are Involved in DCE-induced Migration of U87 Glioma Cells

**Figure 5:**
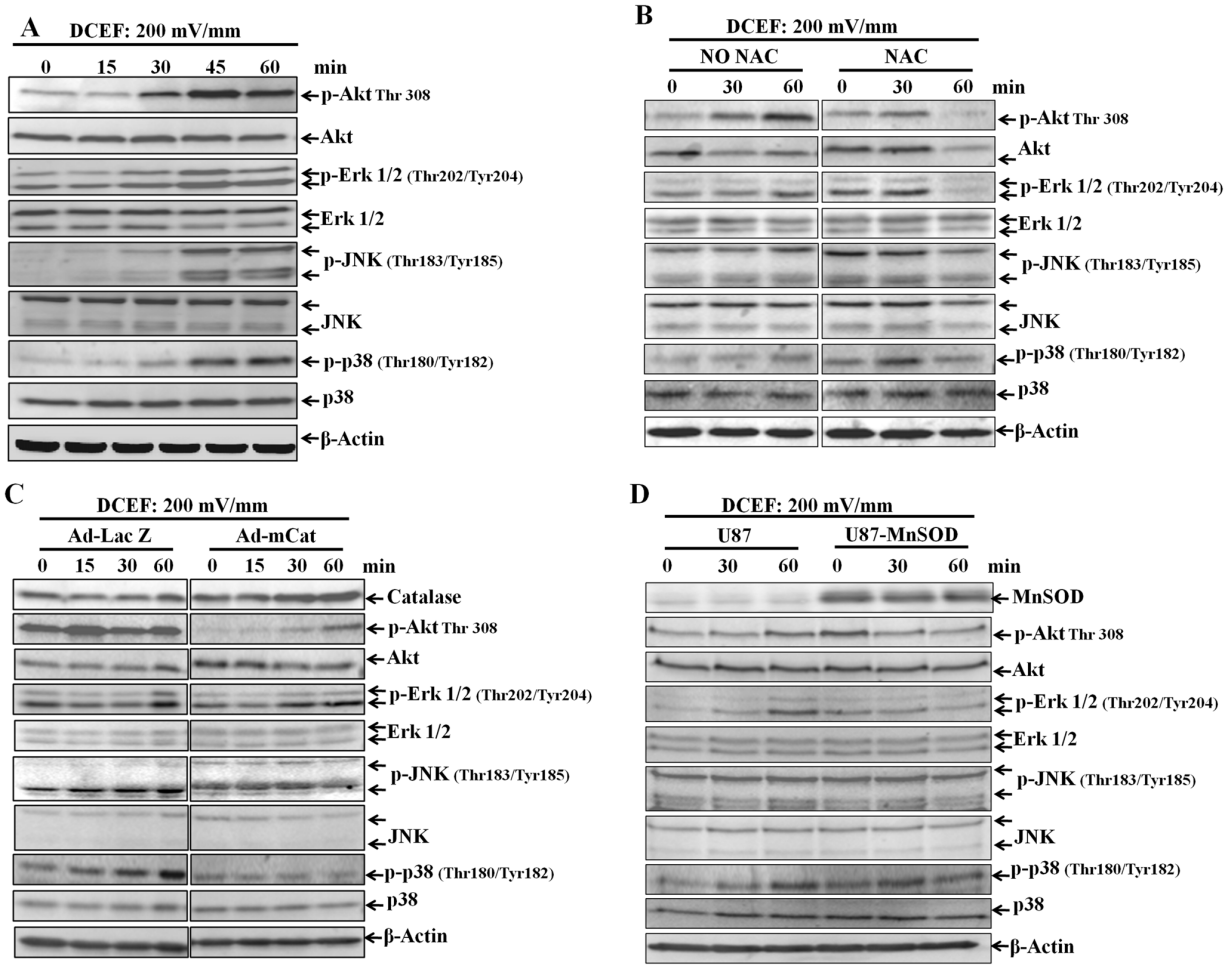
DCEF-stimulated activation of Erk1/2, JNK, and p38 in U87 glioma cells. U87 glioma cells were stimulated with 200 mV/mm DCEF for 0, 15, 30, 45, or 60 minutes (A). The cell lysate was probed with antibodies specific for p-Akt, total Akt, p-Erk1/2, total Erk1/2, p-JNK, total JNK, p-p38, and total p38. Activation of Erk1/2, JNK, and p38 in U87 glioma cells with or without 10 mM NAC pretreatment were stimulated with 200 mV/mm DCEF for 0, 30, and 60 minutes (B). Activation of Erk1/2, JNK, and p38 in U87 glioma cells with or without overexpression of mCat were stimulated with 200 mV/mm DCEF for 0, 30, and 60 minutes (C). Activation of Erk1/2, JNK, and p38 in U87 glioma cells with or without overexpression of MnSOD were stimulated with 200 mV/mm DCEF for 0, 30, and 60 minutes (D). Data presented are a typical representation of a duplicated experiment.

Previous studies have shown that the activation of MAPK and PI3K is essential for cell migration [33], [34], [35], [36], and that an EF can stimulate MAPK in several cell types [4], [37]. To examine if cell signaling pathways are activated in DCEF-stimulated cells, we examined the activation status of Akt, as well as the mitogen-activated protein kinases Erk1/2, JNK, and p38 based on quantifying the intensity of the Western-blots. DCEF (200 mV/mm) increased overall activation of p-Akt, p-Erk 1/2, p-JNK, and p-p38. As shown in Figure 5A, DCEF stimulation of Akt, Erk1/2, JNK, and p38 phosphorylation, which signified activation of the proteins, peaked after 30–45 minutes, and maintained this high level for 60 minutes post-treatment.

We next pre-treated cells with 10 mM NAC for 30 minutes before subjected them to DCEF stimulation. As shown in Figure 5B, the presence of an antioxidant almost completely prevented the activation of Akt, Erk1/2, JNK, and p38. Although the activation of JNK and p38 was also inhibited by over expression of CAT, the activation of Akt and Erk1/2 was not affected (Fig. 5C). However, overexpression of MnSOD almost completely prevented the activation of Akt, Erk1/2, JNK, and p38 (Fig. 5D). These results indicated that the activation of Akt and Erk1/2 by superoxide are involved in DCE-induced migration of U87 glioma cells.

### Superoxide-mediated Electrotaxis Depends on the Activation of Akt and Erk1/2

**Figure 6:**
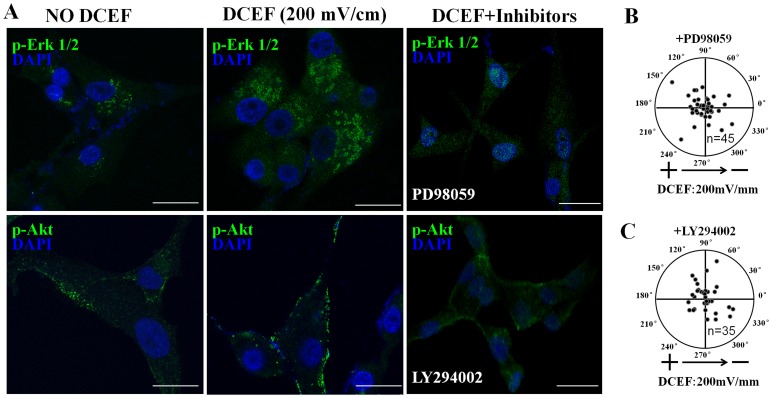
Superoxide-mediated electrotaxis depends on the activation of Akt and Erk1/2. DCEF would induce the phosphorylation of Erk1/2 and Akt, and the activation of Erk1/2 or Akt were reduced by 20 µM PD98059 (inhibitor of Erk) or 20 µM LY294002 (inhibitor of Akt) respectively (A). DCEF-induced directional migration of U87 cells was also significantly decreased by PD98059 (B) or LY294002 (C). Bar = 25 µm.

To investigate whether Akt and Erk1/2 activation are required in DCEF-induced directional migration, we used PD98059, a specific inhibitor of ERKs, and LY294002, a specific inhibitor of Akt. As illustrated in Figure 6A, DCEF would induce the phosphorylation of Erk1/2 and Akt, and the activation of Erk1/2 or Akt were reduced by 20 µM PD98059 or 20 µM LY294002 respectively. Moreover, the DCEF-induced directional migration of U87 cells was also significantly decreased (Fig. 6B and C). The using of PD98059 or LY294002 also decreased the activation of Erk1/2 or Akt in U251 cells and the directional migration (data not shown). This suggested that the DCEF-induced superoxide mediated electrotaxis are dependent on the activation of Erk and Akt.

## Discussion

Glioma cells exhibit the marked ability to invade normal brain tissue, and therefore an understanding of the mechanisms underlying glioma cell invasion will help to improve existing therapies and develop novel therapeutic strategies. In the central nervous system, DCEFs are involved in the modulation of neurogenesis, axon guidance, and nerve growth, and has been explored as a potential therapeutic strategy for the treatment of brain damage [3], [4], [6], [7], [8], [9], [11], [12], [14], [30], [31]. Several types of cells migrate directionally to either the anode or cathode when exposed to a DCEF [38]. Therefore, we reasoned that DCEF may also provide guidance cues to mediate glioma invasion in the brain. The present study is the first to show that glioma cells (U87, C6 and U251) but not astrocytes are directed by DCEF and migrate towards the cathode at a field-strength of 200 mV/mm *in vitro*. Although this phenomenon should be further elucidated *in vivo*, the field strength demonstrated in this study could be easily achieved around the glioma of the brain, especially in cases where abnormal discharges are stimulated by the glioma itself. This could also explain why anti-epileptic drugs, such as Valproate [39], which might exhibit certain anti-glioma activity through inhibit the abnormal discharges of brain. However, the different effects of DCEF on glioma cells and astrocytes are worth paying more attention to.

Some signaling mediators of DCEF-directed cell migration have been recently identified. MAPK and PI3K are key molecules that mediate an electrotactic response. When cells polarize and migrate directionally in response to chemoattractant gradients, the activation of MAPK and PI3K polarizes to the leading edge [40], [41]. Physiological electric fields rapidly and specifically activate PI3K and MAPK signaling pathways, and the activation of those signaling pathways is distributed and polarized in the direction of cell migration [14], [29], [42]. Our observation that DCEF can induce the activation of Akt, Erk1/2, JNK, and p38 in U87 glioma cells is in agreement with these results. However, the factors that bridge the collected electrical stimulation, and the activation of intracellular signaling pathways have yet to be identified [4].

ROS, including superoxide (O_2_^•−^), hydroxyl radical (^•^OH), and hydrogen peroxide (H_2_O_2_), are generated by the mitochondrial respiratory chain and are traditionally thought to be toxic by-products of cellular metabolism. ROS can be regulated by intrinsic antioxidant enzymes. SOD can catalyze superoxide into hydrogen peroxide, which can then be catalyzed by CAT [43]. Several studies originally suggested that ROS are associated with aging [44], cancer [45], [46], and degenerative diseases [47]. However, this definition has been greatly expanded, and it is now widely accepted that ROS play important roles in the modulation of signaling pathways in invasion and migration as well [23], [45], [48], [49]. ROS can regulate the signaling pathways that are responsible for lamellipodia formation, actin cytoskeleton remodeling, focal adhesion turnover, and contraction of the cell body through oxidation and activation of protein tyrosine phosphatases, protein tyrosine kinases, receptor tyrosine kinases, and transcription factors [50]. Our present study demonstrated that an EF of 200 mV/mm dramatically increased hydrogen peroxide and superoxide generation by up to 2-3-fold in glioma cells, and induced significant activation of Akt, Erk1/2, JNK, and p38. Moreover, the addition of NAC or the overexpression of SOD almost completely prevented the activation of these four signaling proteins. These results indicate that the generation of ROS was critical for DCEF-induced activation of MAPKs and PI3K in U87 glioma cells. The directional cathode migration of glioma cells was abolished by the application of NAC or overexpression of SOD, but was not affected by the overexpression of CAT. These results suggest that the generation of superoxide, but not hydrogen peroxide, may play a role in the DCEF-induced directional migration of glioma cells. Together, these novel findings provide further evidence of the role of superoxide in bridging DCEF stimulation and the activation of intracellular signaling pathways.

Furthermore, we found that the activation of JNK and p38 was also inhibited by overexpression of CAT, but Akt and Erk1/2 activation was not affected. Because the overexpression of CAT can inhibit the activation of JNK and p38, but cannot inhibit the directional migration of glioma cells, JNK and p38 may not play a role in this response. Importantly, the activation of Akt and Erk1/2 plays critical role in the response of glioma cells to DCEF, since the using of specific inhibitor can decrease the activation of Erk1/2 or Akt and the directional migration.

**Figure 7:**
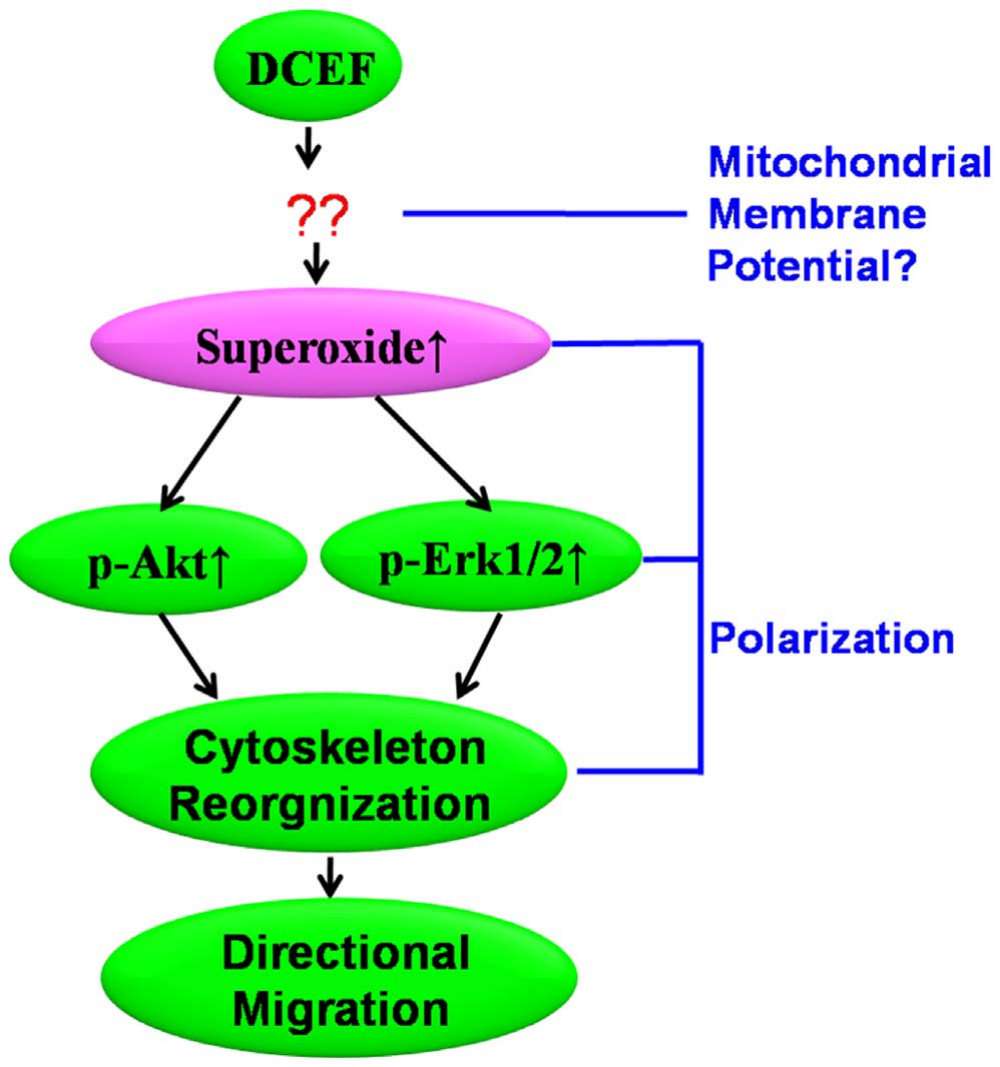
Schematic diagram of superoxide acting as a “bridge” between the extracellular electric stimulation and the intracellular signals in electrotaxis. DCEF induces the generation of superoxide. The superoxide then activates the Erk1/2 and Akt. The activation of these signaling pathways might be critical for the rearrangement and polarization of the cytoskeleton, which gears up the directional migration.

In summary, our results suggest that superoxide play a critical role in DCEF-induced directional migration of glioma cell through the regulation of Akt and Erk1/2 activation. We found that the application of DCEF can induce directional migration of U87, C6 and U251 glioma cells to the cathode, and induce intracellular ROS production. This study also provides evidence that superoxide may mediate the directional migration of these cells in response to DCEF. Furthermore, our findings indicate that the activation of Akt and Erk1/2 by superoxide is involved in this process (Fig. 7). Taken together, our studies provided evidences that the superoxide is at least one of the “bridges” coupling the extracellular electric stimulation to the intracellular signals during DCEF-mediated cell directional migration.
